# Five tips to guide beginners and young general physicians on writing clinical image reports

**DOI:** 10.1002/jgf2.569

**Published:** 2022-07-15

**Authors:** Kosuke Ishizuka, Shun Yamashita, Shinichi Katsukura, Hiroki Matsuura

**Affiliations:** ^1^ Department of General Medicine Chiba University Hospital Chiba Japan; ^2^ Department of General Medicine Saga University Hospital Saga Japan; ^3^ Department of Diagnostic and Generalist Medicine Dokkyo Medical University Mibu Japan; ^4^ Department of General Internal Medicine Okayama City Hospital Okayama Japan

## Abstract

We propose the following five tips as important processes for writing clinical image reports: select a suitable case for the clinical image report; take appropriate images; select a journal for submission; prepare models of clinical image reports; and create templates for structuring clinical image reports in advance. We hope that these five tips will help beginners and young general physicians write clinical image reports.

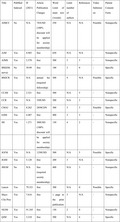

To the Editor,

Among case reports, clinical image reports are relatively easy to write because of the short word limit and the restricted focus of discussion. However, the number of clinical image reports of Japanese generalists published in peer‐reviewed journals is extremely scarce.^1^ The various barriers may exist to write clinical image reports.^1^, ^2^, ^3^ The authors with much experience in publishing articles on clinical image reports conducted interactive interviews and a narrative review of literatures. Consequently, we propose five tips to help beginners and young general physicians write clinical image reports. Furthermore, we provide a table of candidate journals for clinical image reports submission.

### Tip 1: Select a suitable case for the clinical image report

A clinical image report is often written for cases with images directly related to diagnosis and notable characteristic images. In addition to such “impact” cases, “premier” cases with reporting significance of cases or diseases other than imaging findings are suitable for clinical image reports, even if the images do not have a visual impact.

### Tip 2: Take appropriate images

To take appropriate images for acceptance, it should be considered to take 10 to 15 photographs for each finding from different angles, and use a white or pale gray background and avoid shadows.^4^ The videographer should not move when recording the moving physical findings.

### Tip 3: Select a journal for submission

Because general physicians cover a wide range of diseases and practice settings, there are a variety of suitable journals. Although a list of journals suitable for case reports in the area of general medicine has been reported,^5^ we propose the version focused solely on clinical image reports and added several important items (Table 1).

**TABLE 1:** The target journals for clinical image reports written by Japanese generalists

Title	PubMed indexed	IF (2021)	Article publication charges	Word limit of main text (/words)	Limit number of authors	References	Submission	Patient consent form
AIMCC	No	N/A	743USD (100% discount will be applied for society membership)	250	N/A	N/A	Possible	Nonspecific
AJM	Yes	4.965	free	650	N/A	N/A	‐	Nonspecific
AJMS	Yes	2.378	free	500	3	3	‐	Nonspecific
BMJ(Minerva)	No	39.89	free	100	2	0	‐	Specific
BMJCR	Yes	N/A	annual fee (required fellowship)	500	4	N/A	Possible	Specific
CCJM	Yes	2.321	free	500	N/A	5	‐	Nonspecific
CCR	Yes	N/A	350USD	200	N/A	2	‐	Nonspecific
CMAJ	Yes	8.262	free	300	3	3	Possible	Specific
EJIM	Yes	4.487	free	400	3	3	‐	Nonspecific
IM	Yes	1.271	300USD (100% discount will be applied for society membership)	150	4	2	‐	Nonspecific
JGFM	Yes	N/A	1250USD	500	N/A	5	Possible	Specific
JGIM	Yes	5.128	free	200	3	N/A	‐	Nonspecific
JHGM	No	N/A	free (required society membership)	400	N/A	3	‐	Nonspecific
Lancet	Yes	79.321	free	300	N/A	0	Possible	Specific
Mayo Clin Proc	Yes	7.616	free	1 page in the print publication	3	N/A	‐	Nonspecific
NEJM	Yes	91.245	free	150	2	0	‐	Nonspecific
QJM	Yes	3.210	free	500	N/A	6	‐	Specific

Abbreviations: AIMCC, Annals of Internal Medicine Clinical Cases; AJM, American Journal of Medicine; AJMS, American Journal of Medical Sciences; BMJ, British Medical Journal; BMJCR, British Medical Journal Case Reports; CCJM, Cleveland Clinic Journal of Medicine; CCR, Clinical Case Reports; CMAJ, Canadian Medical Association Journal; EJIM, European Journal of Internal Medicine; IF, Impact Factor; IM, Internal Medicine; JGFM, Journal of General and Family Medicine; JGIM, Journal of General Internal Medicine; JHGM, Journal of Hospital General Medicine; Mayo Clin Proc, Mayo Clinic Proceedings; NEJM, New England Journal of Medicine; QJM, Quarterly Journal of Medicine. [Correction added on August 4, 2022, after first online publication: In Table 1, "2850CDN" was changed to " free" in the row starting with “CMAJ”]

### Tip 4: Prepare models of clinical image reports

The format of clinical image reports differs across journals. It is therefore important to obtain several model reports, which can be detected as follows: refer to previous clinical image reports by supervisors or acquaintances; search for clinical image reports on the website of the target journal; or search PubMed or Google Scholar for clinical image reports about the same diseases.

### Tip 5: Create templates for structuring clinical image reports in advance

The discussion consists mainly of the following three elements: overview of the disease/conditions; findings of the image; and instructive message. The reports of cases with “premier” result in the addition of the fourth premier element. These are required to be mentioned with their supporting references. In the element of overview, a simple explanation of the concept or etiology of the disease or conditions and epidemiology are mentioned. In the “premier” element, the reportable significance should be indicated such as common diseases with an uncommon onset, symptoms, and clinical course. In the element of the findings of the images, the representative findings of the disease should be mentioned. Finally, in the element of the instructive message, the lessons learned from the case should be summarized for future cases.

We hope that these five tips will help beginners and young general physicians write clinical image reports.

### AUTHOR CONTRIBUTION

All authors had access to the data and a role in writing the manuscript.

### CONFLICT OF INTEREST

None.

REFERENCES1

Kenzaka
T
, 
Kamada
M
. Barriers to preparation of case reports among Japanese general practitioners. Saudi J Med Med Sci. 2020;8:239–40.3295251810.4103/sjmms.sjmms_604_19PMC74856602

Shikino
K
, 
Watari
T
, 
Tago
M
, 
Sasaki
Y
, 
Takahashi
H
, 
Shimizu
T
. Five tips on writing case reports for Japanese generalists. J Gen Fam Med. 2021;22:111–2.3371779110.1002/jgf2.395PMC79213313

Yamashita
S
, 
Sasaki
Y
, 
Miyagami
T
, 
Kondo
T
. Five tips to help young, non‐native speakers of English write reports of cases presented at academic conferences. J Hosp Gen Med. 2022;4:36–9.4

Muraco
L
. Improved medical photography: key tips for creating images of lasting value. JAMA Dermatol. 2020;156:121–3.3189542710.1001/jamadermatol.2019.38495

Tago
M
, 
Watari
T
, 
Shikino
K
, 
Sasaki
Y
, 
Takahashi
H
, 
Shimizu
T
. To which journal should generalists submit a clinical case report?
J Hosp Gen Med. 2020;2:99–103.
